# Stop and Change: Inhibition and Flexibility Skills Are Related to Repetitive Behavior in Children and Young Adults with Autism Spectrum Disorders

**DOI:** 10.1007/s10803-015-2473-y

**Published:** 2015-06-05

**Authors:** Mandy A. L. Mostert-Kerckhoffs, Wouter G. Staal, Renske H. Houben, Maretha V. de Jonge

**Affiliations:** Department of Child and Adolescent Psychiatry, Brain Center Rudolf Magnus, University Medical Center Utrecht, Heidelberglaan 100, 3584 CX Utrecht, The Netherlands; Donders Centre for Neuroscience, Radboud University Nijmegen Medical Centre, Reinier Postlaan 12, 6525 GC Nijmegen, The Netherlands; Karakter Centre for Child and Adolescent Psychiatry, Reinier Postlaan 12, 6525 GC Nijmegen, The Netherlands; Department of Medical Psychology, University Medical Center Amsterdam, Amsterdam, The Netherlands; Rijndam Rehabilitation, Dordrecht, The Netherlands

**Keywords:** Autism spectrum disorders, Repetitive behaviors, Inhibition, Flexibility, Auditory information

## Abstract

Cognitive control dysfunctions, like inhibitory and attentional flexibility deficits are assumed to underlie repetitive behavior in individuals with autism spectrum disorders (ASD). In the present study, prepotent response inhibition and attentional flexibility were examined in 64 high-functioning individuals with ASD and 53 control participants. Performance under different task conditions were tested both in response to visual and auditory information, and requiring a motor or verbal response. Individuals with ASD showed significant more control dysfunctions than typically developing participants on the auditory computer task. Inhibitory control and attentional flexibility predicted RRB in everyday life. Specifically, response inhibition in reaction to visual information and task switching in reaction to auditory information predicted motor and sensory stereotyped behavior.

## Introduction

Routines, rituals and repetitive patterns of behavior are among the core symptoms of autism spectrum disorders (ASD; APA 2000). Cognitive control dysfunctions, such as impaired attentional flexibility and inhibitory deficits, are assumed to underlie inflexible behavior in individuals with ASD. However, replication has proven to be difficult (for reviews see Geurts et al. 2009; Hill 2004; Russo et al. 2007). This inconsistency in findings might be caused by different methodological issues, such as differences between studies in tasks or task conditions (Brunsdon and Happé 2014; Williams and Jarrold 2013), but also by heterogeneity of the ASD phenotype and by the variation of ASD characteristics over the span of life. Clinically, this theory is compelling because it may lead to interventions that focus on improving attentional flexibility and inhibition skills. Indeed, deficits in both attentional flexibility and response inhibition have been demonstrated in children, adolescents and adults with ASD.

Even more complicating is the fact that correlations between inflexible or repetitive behavioral patterns and attentional flexibility shows mixed results. In a sample of high-functioning children with ASD positive correlations were found between the Restricted and Repetitive Behavior (RRB) domain score on the ADI-R (Autism Diagnostic Interview-Revised version; Lord et al. 1994) and the error rate on a set-shifting task (Yerys et al. 2009). In contrast, this correlation was not found in a prior study with a large sample of ASD individuals with a broad age range (Ozonoff et al. 2004). In adolescents with ASD, positive correlations were found between the number of perseverative responses on the WCST and the RRB scores on the ADI-R and ADOS (Lord et al. 2000), but not with other measures of RRB (South et al. 2007). Similarly, no correlation was found between shifting performance on an adapted WCST task and a RRB questionnaire in ASD children and adolescents (Dichter et al. 2010). However, Lopez et al. (2005) found ASD adults with attentional flexibility difficulties to show high levels of RRB in everyday life.

Several explanations for these inconsistencies have been proposed. First, cognitive control tasks rarely measure ‘pure’ functions. Performance usually depends on multiple functions such as (motor) response speed, basal attention, and error processing in addition to inhibitory control and attentional flexibility. Since deficits in these areas are often found in ASD (i.e. Goldberg et al. 2005; Schmitz et al. 2007; Shafritz et al. 2008; Steele et al. 2007; Stoet and López 2011; Sturm et al. 2004; Verté et al.^2006^; Williams et al. 2005), it is important to control for these functions in order to draw conclusions about inhibitory or flexibility deficits in ASD. Second, task conditions like degree of open-endedness, task structure and administration have been found to influence task performance (Teunisse et al. 2001; van Eylen et al. 2011; White et al. 2009). Third, some tasks require a verbal response while other tasks require motor responses. Fourth, across different studies, flexibility tasks have been based on visual cues (e.g. requiring a reaction to visual stimuli) and to our knowledge it is unknown whether individuals with ASD experience cognitive control difficulties to an equal extend when auditory stimuli are used. This may be highly relevant since visual information processing may be a preferred cognitive style in some ASD patients (Depape et al. 2012), while at the same time audio-visual integration seems to be impaired in ASD (Kunda and Goel 2011). Fifth, developmental effects have to be taken into account. For instance, executive functioning impairments in ASD appear to be less pronounced in adults than adolescents, while visuomotor abnormalities are present in both adolescents and adults (Sachse et al. 2013).

The purpose of the present study was to compare the ability to inhibit a prepotent response and attentional flexibility between high-functioning children or young adults with ASD and typically developing individuals. We took care to assess inhibitory control and attentional flexibility in a systematic way, controlling for basal attention and response speed. Computer tasks with visual and auditory stimulus conditions were used requiring motor responses, in addition to more classic flexibility tasks requiring a verbal response or pattern drawing. It was expected that individuals with ASD experience more difficulties than control individuals with prepotent response inhibition and attentional flexibility, when basal attention and response speed is controlled for. We hypothesized that these difficulties would be found to an equal extend in the auditory and visual flexibility tasks. We expected a developmental change in these high-functioning individuals and therefore expected performance to increase with age in both groups, but possibly more so in the control group than in the ASD group. In addition, the association between inhibitory control or attentional flexibility difficulties and RRB in daily life was investigated. We expected these cognitive control functions to be related to RRB in everyday life.

## Methods

### Participants

Sixty six high-functioning individuals with ASD and 56 control participants participated in this study. Both groups consisted of a subgroup of children (age 8–13) and adolescents/young adults (age 16–26). Individuals in the ASD group were recruited from the department of psychiatry at the University Medical Center Utrecht, control participants through local schools. Exclusion criteria were: significant medical disorders, seizures or a history of brain injury, color blindness, FSIQ below 70 (determined by the short form of the Wechsler scales (four subtests: vocabulary, similarities, block design, object assembly; Wechsler 2005a, b) or SRS total score outside the normal range for the control participants. Participants in the control group were included when there was no indication of an ASD or other developmental disorder in the subjects or their first-degree relatives by telephonic screening. To confirm the absence of ASD-like behavior, the Social Responsiveness Scale was administered (SRS; Constantino and Todd 2000).

Two children from the ASD group were excluded because of behavioral difficulties during task administration and consequent scores on the cognitive tests above four SD from average. Two young adults were excluded from the control group, because of scores in the severe or clinical range on the SRS. One child was excluded from the control group because of color blindness. The final ASD group comprised 32 children and 32 young adults with a clinical diagnosis of Autism (*n* = 25), Asperger syndrome (*n* = 13) or PDD-NOS (*n* = 26) made by an expert child- and adolescent psychiatrist. The clinical diagnoses were conformed with the ADI-R (Lord et al. 1994), or the ADOS (Lord et al. 2000) and in most cases with both instruments. ADI-R and ADOS were administered by experienced and certified examiners. The ADI-R and ADOS scores of two patients could not be obtained. Seventeen participants (13 in the child group and four in the young adult group) used medication, including stimulants, atypical antipsychotics or a combination of both. For ethical reasons, medication was not withheld prior to testing. The control group comprised 27 children and 26 adolescents/young adults. None of the controls were on medication.

The groups were stratified for sex, age and FSIQ. Ethical approval for the study was obtained and all participants or their parents, if appropriate, gave written informed consent.

### Measures/Materials

Participants were screened for color blindness with the Hardy–Rand–Rittler Pseudo isochromatic Plates fourth edition (Hardy et al. 1954).

### Cognitive Control

Cognitive control was measured by means of three tasks that all comprise of a baseline condition, inhibition of a prepotent response condition and attentional flexibility condition. Two tasks are part of a computerized test battery (the Amsterdam Neuropsychological Tasks; De Sonneville 1999). These computer tasks have a visual stimulus condition (SSV) and auditory stimulus condition (SSA) and require a motor response. The third task was the Color Word interference test (CW) from the D-KEFS battery (Delis et al. 2001). The CW is an experimenter assessed visual Stroop test, requiring verbal responses. In addition, the Design Fluency test (DF) of the D-KEFS was administered. This task also has three conditions; two baseline conditions and one attentional flexibility condition, but no inhibition condition. The DF is a paper-and-pencil task designed to measure nonverbal fluency and attentional flexibility. Each of every task condition was preceded by an instruction and a practice session. Participants were encouraged to react as quickly and accurately as they could. Dependent variables were response time (in ms for computer tasks, in sec for CW) and total number of errors. Dependent variables of the DF were total number of patterns produced and total number of errors.

#### Shifting Attentional Set-Visual (SSV)

In all conditions, a horizontal bar consisting of ten grey squares is presented at the center of a laptop computer screen. Responses were required between 150 and 5000 ms (otherwise a trial was replaced). The task is self-paced and has a 250 ms post-response interval.

*Condition 1: baseline speed and accuracy* (ten practice trials, 40 experimental trials).

A green colored square moved across the bar in a random direction, either to the right or left. Participants were asked to respond in a spatially *compatible way* by pressing the response button that corresponded to the direction in which the stimulus moved.

*Condition 2: inhibition of a prepotent response* (ten practice trials, 40 experimental trials).

A red colored square moved across the bar in a random direction. Participants had to respond in a spatially *incompatible way* by pressing the response button that corresponded *opposite to* the direction in which the stimulus moved.

*Condition 3: attentional set shifting* (16 practice trials, 80 experimental trials).

The color of the moving square alternated in a random fashion between green and red. Both the direction and color of the square were unpredictable. The color of the square simultaneously changed, as the square moved one position. When the square was green, a compatible response was required (as in task 1). When the square was red, an incompatible response was required (as in task 2).

#### Shifting Attentional Set-Auditory (SSA)

The task resembles the SSV task but auditory stimuli are used. The auditory stimuli had a duration of 100 ms and a post response interval of 1200 ms.

*Condition 1: baseline speed and accuracy* (ten practice trials, 40 experimental trials).

The computer presented a low-pitched tone (200 Herz) either once or twice. Participants were asked to respond in a *compatible way* by pressing the response button that corresponded to the sound: once, when one tone was presented; twice, when two tones were presented.

*Condition 2: inhibition of a prepotent response* (ten practice trials, 40 experimental trials).

The computer presented a high-pitched tone (400 Herz) either once or twice. Participants had to respond in an *incompatible way* by pressing the response button that corresponded *opposite to* what was heard: once, when two tones were presented; twice, when one tone was presented.

*Condition 3: attentional set shifting* (16 practice trials, 80 experimental trials).

Both pitch and quantity of the tone were unpredictable. The pitch and quantity simultaneously changed. When a low-pitched tone was presented, a compatible response was required (as in task 1). When a high-pitched tone is presented, an incompatible response was required (as in task 2).

#### Color Word Interference Test (CW)

In addition to the conditions of the traditional Stroop test, two extra tasks are designed. In the first task, subjects have to name colors as quickly as possible. This task was not used in this study. The other condition is designed to measure attentional switching. In each condition, 50 stimuli are displayed in five rows of ten stimuli on a card. Conditions 3 and 2 are related to the first condition in order to measure inhibition and set shifting.

*Condition 1: baseline speed and accuracy*

Participants were asked to read color names printed in black ink.

*Condition 2: inhibition of a prepotent response*

The color names are printed in an incompatible ink color. Participants were asked to name the ink color while suppressing the tendency to read the word, which requires inhibiting the automatic reading response.

*Condition 3: attentional set shifting*

Participants were instructed to alternate between reading the color names and naming the discordant ink colors. Half of the words are presented in a box. These words have to be read, while the ink color has to be named with words that do not appear in a box.

#### Design Fluency Test (DF)

In each condition, a number of dots had to be connected by drawing four straight lines to complete as many unique designs as possible in 1 min. The dots are presented in boxes arranged in five rows of seven boxes.

*Condition 1: baseline speed and fluency*

Each box contained five filled (i.e. black) dots. Participants were asked to connect the dots.

*Condition 2: baseline speed and fluency*

Each box contained five filled and five empty dots. Participants only had to connect the empty dots.

*Condition 3: fluency and attentional set shifting*

Each box contained five filled and five empty dots. Participants had to alternate between filled and empty dots.

### Repetitive Behavior

#### Autism Diagnostic Interview-Revised (ADI-R)

The ADI-R is a standardized, semi-structured parent interview designed to obtain detailed descriptions of ASD symptoms both currently and during early development. The ADI-R focuses on communication skills, social development and play, repetitive and restricted behaviors and general behavior problems. The ADI-R has good interrater reliability (Cicchetti et al. 2008), test–retest reliability (Hill et al. 2001) and validity (De Bildt et al. 2004).

A factor analysis revealed that two different factors underlie the RRB domain (Cucarro et al. 2003). The Insistence on Sameness (IS) factor reflects resistance to change while the Repetitive Sensory and Motor Behaviors and interests (RSMB) factor can be described as lower order motor and sensory repetitive behavior. For the purpose of this study the total “current” scores of the RRB domain and the two underlying factors were used for the analyses of the correlation between cognitive performance and everyday life behavior.

#### Autism Diagnostic Observation Scale (ADOS)

The ADOS is a semi-structured interactive assessment designed to observe behavior indicative of autism involving social behavior, communicative functioning, and restricted or repetitive behavior. The ADOS has excellent interrater reliability, internal consistency and test–retest reliability on item, domain and classification levels for ASD and non-spectrum disorders (Lord et al. 2000). In the present study the total score on the RRB domain was used to investigate the association between cognition and behavior.

#### Autism Questionnaire (AQ)

The AQ is a 50-item questionnaire designed to measure the degree of autistic traits on a continuum from normality to autism, shown by a person of normal intelligence. It consists of five subscales: ‘social skills’, ‘communication’, ‘imagination’, ‘attentional switching’ and ‘attention to detail’. The AQ-Adult Version and the AQ-adolescent Version (12–16 years) depend on self-report. Both versions have good to excellent test–retest reliability and reasonable to high internal consistency (Baron-Cohen et al. 2001, 2006). The AQ-Children’s version is a parent-report questionnaire that aims to quantify traits in children 4–11 years old. This instrument has good test–retest reliability and high internal consistency (Auyeung et al. 2008). In the present study the subscales ‘attentional switching’ and ‘attention to detail’ were used to study the association between cognition and behavior.

### Data Collection Procedure

The data were collected at the department of child and adolescent psychiatry. All participants were individually tested in a quiet room.

### Missing Values and Outliers

Technical problems with the computer lead to missing values (MV) in three cases. The MV did not exceed 5 % and the percentage was balanced over the ASD and control groups. Outliers were detected using SPSS (version 15). Data exceeding 4sd from the mean were excluded from analyses; two children in the ASD group were excluded for this reason.

### Statistical Analysis

Statistical analyses were performed using SPSS-20^®^. To reduce the risk of type I errors because of multiple testing, a *p* value <.01 was deemed significant. A Chi square analysis was conducted to test whether the two groups differed relative to gender. Age and FSIQ were analyzed using a *T* test for independent groups.

Repeated measures analyses were performed to test for differences between the ASD and the control group in RT or errors in the tasks separately. Reaction times of the SSA, total patterns of the DF and errors of the CW, DF and SSA were normally distributed. Reaction times of the CW, SSV and errors of the SSV were normalized by applying a natural log inverse-transformation. For all tests, degrees of freedom were corrected using Greenhouse-Geisser estimates of sphericity. The repeated measure ‘Condition’ had three levels (baseline, prepotent response inhibition, attentional flexibility) and the between subject factors were ‘Group’ (ASD vs controls) and ‘Age-group’ (8–12 vs 16–26).

In addition, the extent to which inhibition and attentional flexibility predict RRB in daily life was studied by performing six linear and stepwise multiple regression models. The RRB total domain score, IS factor and RSMB factor of the ADI-R; the RRB total domain score of the ADOS and the subscale ‘attention switching’ of the AQ were used as dependent measures for repetitive behavior. The predictor variable in the enter models was age. For all measures an inhibition and switch cost variable was calculated by subtracting the RT on the baseline task from the RT in the inhibition and flexibility conditions. The variables entered into the stepwise models were FSIQ, inhibition and switch cost on reaction times and errors. In order to examine whether cognitive control performance is associated with the severity of ASD symptomatology, correlations between the SRS total score and cognitive control performance was calculated.

## Results

### Descriptive Statistics

Demographic characteristics of the participants are presented in Table 1. In both the child and young adult group, the individuals with ASD and controls did not differ relative to gender, age and FSIQ.

**Table 1:** Age, sex, total intelligence quotient (TIQ) and handedness of the participants with autism spectrum disorders (ASD) and controls by age group

	8–13			16–26		
	ASD (*n* *=* *32)*	Controls (*n* *=* *27)*	*p*	ASD (*n* = 32)	Controls (*n* = 26)	*p*
Age in years (M/SD)	11.3 (1.4)	11.0 (1.2)	.29	20.5 (3.2)	20.7 (2.1)	.79
Sex (% male)	78	78	*.97*	78	81	*.81*
TIQ (M/SD)	110.6 (16.4)	112.5 (14.5)	*.65*	111.1 (18.4)	112.4 (15.4)	*.78*
VIQ (M/SD)	108.6 (16.6)	115.6 (15.8)	.*10*	104.9 (14.8)	102.7 (17.8)	*.61*
PIQ (M/SD)	108.6 (20.6)	105.6 (16.2)	*.54*	116.4 (25.2)	124.5 (19.3)	*.17*
Handedness (*n* right)	27	25		27	23	

Italic values are statistically significant at 0.01

#### Inhibition of Prepotent Responses and Attentional Flexibility

##### ANT: Shifting Attentional Set-Visual (SSV)

**Fig. 1:**
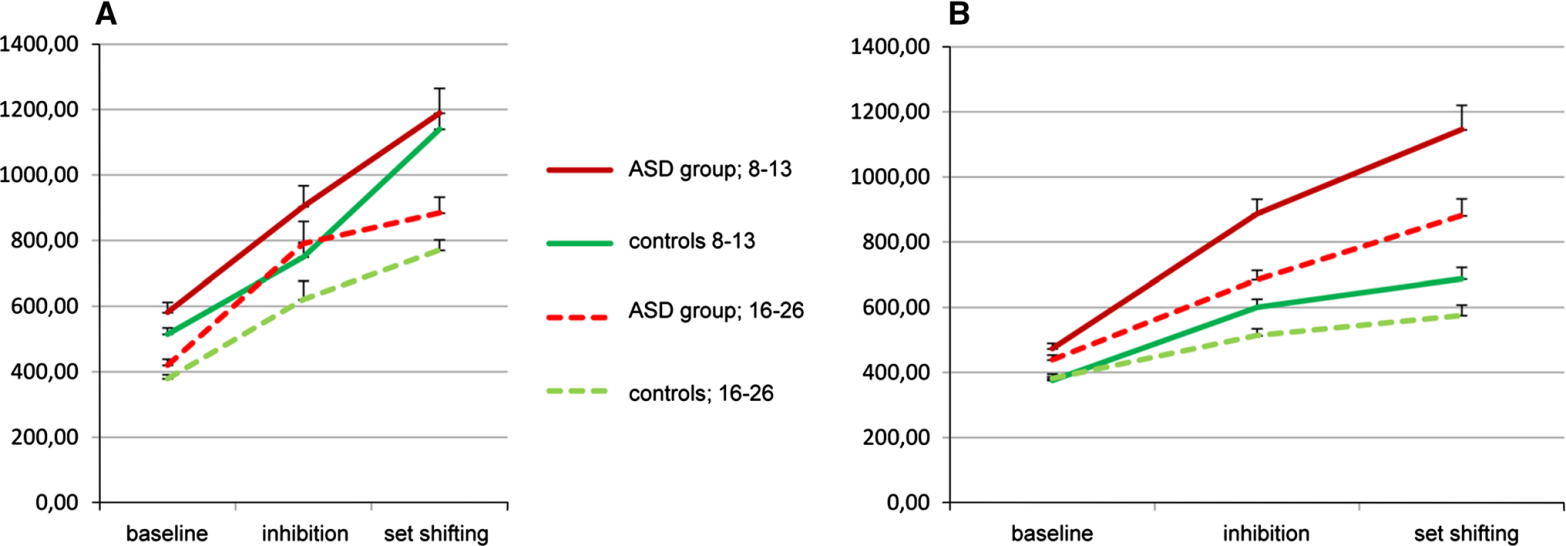
Reaction times on the visual and auditory computer tasks in milliseconds for the ASD-group and controls with age for the baseline, inhibition and attentional flexibility conditions. **a** Visual computertask. **b** Auditory computertask

The results of the analyses are presented in Table 2. Reaction times of both groups on the SSV are presented in Fig. 1. There was no significant three-way interaction Condition × Group × Age-group. A trend was observed for the interaction Condition × Group, although this did not reach significance. Individuals with an ASD did not have extra problems compared to controls with increasingly difficult conditions. The Condition × Age-group interaction was significant. The difference between younger and older participants increased on more difficult conditions. Follow-up contrasts show that this was the case on both the inhibition and flexibility conditions. A significant main effect of Condition on mean reaction time was present, indicating that, as expected, both participants with an ASD and controls were slower on the more difficult inhibition and flexibility conditions.

**Table 2:** Results of the statistical analysis

	SSV			SSA			CW			DF		
	F	*p*	ηp^2^	F	*p*	ηp^2^	F	*p*	ηp^2^	F	*p*	ηp^2^
*Reaction time*												
Condition												
Overall	484.62	<*.0001*		231.72	<*.0001*		1436.01	<*.0001*		54.67	<*.0001*	
2 vs 1	334.94	<*.0001*	.751	383.54	<*.0001*	.774	1459.64	<*.0001*	.928			
3 vs 1	1029.82	<*.0001*	.903	308.42	<*.0001*	.734	1764.91	<*.0001*	.940	31.29	<*.0001*	.217
Condition*group												
Overall	3.66	.03		11.29	<*.0001*		2.11	.12		0.39	.68	
2 vs 1	1.004	.32	.009	24.98	<*.0001*	.182	2.98	.09	.026			
3 vs 1	2.78	.10	.024	14.21	<*.0001*	.113	1.55	.22	.013	0.00	.98	.000
Condition*age												
Overall	12.31	<*.0001*		32.12	<*.0001*		19.18	<*.0001*		2.76	.07	
2 vs 1	15.96	<*.0001*	.126	33.99	<*.0001*	.233	6.91	*.01*	.058			
3 vs 1	19.55	<*.0001*	.150	43.67	<*.0001*	.281	28.74	<*.0001*	.203	2.79	.10	.024
Condition*group*age												
Overall	0.54	.59		1.15	.31		0.36	.70		2.35	.10	
2 vs 1	0.56	.45	.005	2.14	.15	.019	0.03	.87	.000			
3 vs 1	1.01	.32	.009	1.50	.22	.013	0.29	.59	.003	0.00	.96	.000
*Errors*												
Condition												
Overall	19.47	<*.0001*		15.41	<*.0001*		49.64	<*.0001*		12.87	<*.0001*	
2 vs 1	17.23	<*.0001*	.133	19.28	<*.0001*	.147	76.18	<*.0001*	.403			
3 vs 1	34.56	<*.0001*	.236	21.34	<*.0001*	.160	108.19	<*.0001*	.489	22.71	<*.0001*	.167
Condition*group												
Overall	0.28	.76		1.06	.35		0.60	.55		0.56	.57	
2 vs 1	0.45	.51	.004	1.76	.19	.015	0.93	.34	.008			
3 vs 1	0.34	.56	.003	0.01	.97	.000	1.29	.26	.011	0.00	.96	.000
Condition*age												
Overall	1.59	.21		4.30	.02		8.60	<*.0001*		0.11	.89	
2 vs 1	2.70	.10	.024	8.65	.004	.072	17.51	<*.0001*	.134			
3 vs 1	0.14	.71	.001	3.26	.07	.028	2.34	.13	.020	0.14	.71	.001
Condition*group*age												
Overall	0.05	.95		0.34	.69		0.31	.74		0.87	.42	
2 vs 1	0.08	.78	.001	0.52	.47	.005	0.65	.42	.006			
3 vs 1	0.05	.82	.000	0.43	.52	.004	0.27	.61	.002	1.05	.31	.009

Italic values are statistically significant at 0.01

Reaction time s and errors of the inhibition (2) and flexibility (3) conditions on the shifting attentional set-visual test (SSV), shifting attentional set-auditory test (SSA), color word interference test (CW) and design fluency test (DF)

Error analysis revealed no significant three way interaction Condition × Group × Age-group and no significant Condition × Age-Group interaction. The Condition × Group interaction was also not significant; participants with an ASD made no more errors than controls. There was a significant main effect of Condition. Both groups made more errors, as the follow-up show, in the inhibition and flexibility conditions.

##### ANT: Shifting Attentional Set-Auditory (SSA)

Reaction times of both groups on the SSA are presented in Fig. 1. Analysis showed no significant three-way interaction Condition × Group × Age-group. Both the interactions Condition × Group and Condition × Age-group were significant. The difference between younger and older participants increased on more difficult conditions, as follow-up contrasts show, on both the inhibition and flexibility conditions. Importantly, results also indicate that participants with an ASD were disproportionally slower. Follow-up contrasts revealed that this was the case for the inhibition and the flexibility conditions, meaning that inhibition of a prepotent response and flexibility were more difficult. The main effect of Condition on mean reaction time was significant, again indicating as expected, that both participants with an ASD and controls were slower on the more difficult inhibition and flexibility conditions.

Error analysis revealed no significant three way interaction Condition × Group × Age-group, no significant Condition × Group interaction and no significant Condition × Age-group interaction. There was a significant main effect of Condition, indicating that all participants made more errors on the more difficult inhibition and flexibility conditions.

##### D-KEFS: Color Word Interference Test

**Fig. 2:**
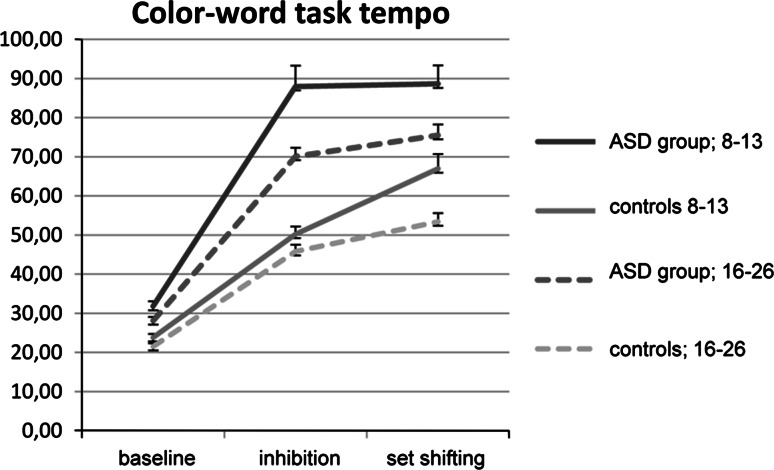
Reaction times on the color word interference test in seconds for the ASD-group and controls with age for the baseline, inhibition and attentional flexibility conditions

Reaction times of both groups on the color word interference test are presented in Fig. 2. Analysis showed no significant three-way interaction Condition × Group × Age-group and no significant Condition × Group interaction. The latter indicates that participants with an ASD were not slower on this test. The interaction Condition × Age-group was significant. Younger participants performed more slowly on the more difficult conditions than on baseline condition. Follow-up contrasts show that this was the case for both the inhibition and flexibility conditions. A significant main effect of Condition on mean reaction time was also present for this test. All participants were slower on the more difficult inhibition and flexibility conditions.

Error analysis revealed no significant three way interaction Condition × Group × Age-group, no significant Condition × Group interaction, but a significant Condition × Age-group interaction. The latter indicates that the difference in errors between younger and older participants increased on the more difficult conditions. Contrasts show that this is only the case in the inhibition condition. There was a significant main effect of Condition, again indicating that more errors were made on the inhibition and flexibility conditions by all participants.

##### D-KEFS: Design Fluency Test

**Fig. 3:**
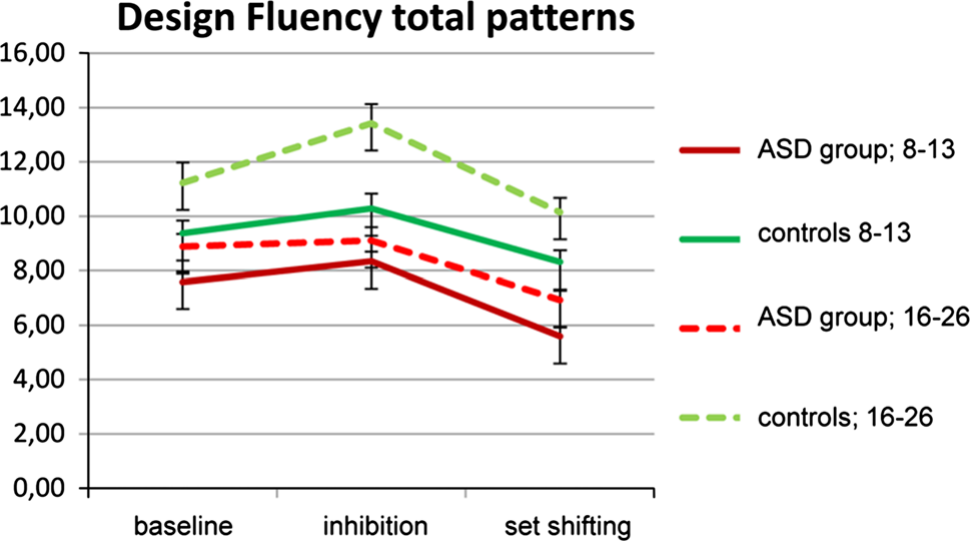
Total patterns created on the design fluency test for the ASD-group and controls with age for the baseline and attentional flexibility conditions

Analysis showed a similar pattern in both reaction time and errors. There was no significant three way interaction Condition × Group × Age-group, no significant Condition × Group interaction and no significant Condition × Age-group interaction. There was a significant main effect of Condition, indicating that, as expected, the flexibility condition was found more difficult by all participants compared to the baseline condition (Fig. 3).

#### Predicting Repetitive Behavior in Everyday Life

##### ADI-R; RRB Total Domain Score, RSMB Factor and IS Factor

All regression coefficients are standardized coefficients. Switch cost total patterns of the DF (*b* = .38, *t* = 2.877, *p* = .006) predicted restricted and repetitive behavior measured with the ADI-R, with a significant proportion of variance in RRB, *R*^2^ = .13, *F*_(2,56)_ = 4.193, *p* = .006. Age (*p* = .266) and IQ (*p* = .080) were not significant independent predictors of RRB.

Inhibition cost accuracy of the SSV (*b* = .43, *t* = 3.521, *p* < .001) predicted RSMB, with a significant proportion of variance *R*^2^ = .25, *F*_(2, 55)_ = 8.78, *p* < .001. Age (*b* = −.34, *t* = −2.778, *p* < .008) also significantly predicted RSMB, but IQ did not (IQ (*p* = .29).

None of the potential independent variables significantly predicted insistence on sameness.

##### ADOS; Total Domain Score Repetitive Behaviors

SSV switch cost accuracy (*b* = .54, *t* = 5.142, *p* < .0001), predicted repetitive behavior measured with the ADOS, with a significant proportion of variance in repetitive behavior scores, *R*^2^ = .41, *F*_(2,57)_ = 18.762, *p* < .0001. Age and IQ were not significant predictors of repetitive behaviors measured with the ADOS with the alpha

level chosen although there was a trend for age (Age *p* = .02, IQ *p* = .774).

##### AQ; Attention Switching

Within the ASD group, only age predicted attention shifting in daily life situations, but none of the cognitive measures. There was a strong negative correlation between age and attention shifting in the ASD group (*r* = −.56, *p* < .0001) indicating that attentional switching skills improved with age in the individuals with ASD, while there was no correlation in the control group (*r* = .07, *p* = .60).

When including the whole sample, SSA inhibition cost speed (*b* = .50, *t* = 5.330, *p* < .0001) predicted attention switching, with a significant proportion of variance, *R*^2^ = .24, *F*_(3,109)_ = 17.606, *p* < .0001. Age (*p* = .907) and IQ (*p* = .43) were not significant independent predictors of attention switching.

##### SRS; Total Score

Cognitive control performance was found to be significantly associated with ASD symptomatology (SSV inhibition cost *r* = −.25, *p* = .046, switch cost *r* = −.34, *p* = .007; SSA inhibition cost *r* = −.34, *p* = .006, switch cost *r* = −.42, *p* = .001; CW inhibition cost *r* = −.44, *p* < .001, switch cost *r* = −.17, ns).

## Discussion

In this study, prepotent response and attentional flexibility skills were compared between high-functioning children and young adults with ASD and typically developing individuals. The results show that response inhibition and attentional flexibility were impaired in ASD subjects, independent of their age. When carefully taking into account effects of basal attention and response speed, significant slower inhibitory control or attentional flexibility was found in the ASD group as a whole, but only in response to auditory information. To our knowledge, prepotent response inhibition and attentional flexibility in response to auditory information has not been studied yet, while controlling for basal attention and response speed.

The data further show the expected developmental change in performance. Younger children were slower and made more errors than young adults when prepotent response inhibition or attentional flexibility was required. This is in line with findings from Happé et al. (2006) who found that children with ASD improved over time on different executive function tests. However, while we expected a stronger developmental change in our controls than in the ASD group, the data did not show this.

A remarkable finding is the difference between visual and auditory tasks of inflexibility. Individuals with ASD needed significantly more time than controls to stop and change on the auditory, but not on the visual set shifting task of the same computer battery. This is an important observation that may have clinical relevance. Inflexible behavior in ASD causes significant impairment and is a burden to patients and caregivers (Mungo et al. 2007). Indeed, cognitive flexibility in ASD appears to be an important predictor of outcome (Berger et al. 2003; Pijnacker et al. 2009). The emphasis on impaired auditory flexibility may reflect preferences for visual information processing in ASD subjects, which is interesting in the light of recent work that relates enhanced visual processing in ASD to underlying neuronal networks (Samson et al. 2012) and studies that show that aberrant visual processing occurs early during development (Pierce et al. 2011). A significant part of information during social situations is transferred verbally. This requires a reaction or switch in behavior to auditory cues, which may, based on the results of the present study, cause specific difficulties in distracting attention from commenced behavior in individuals with ASD. Clinical interventions could focus on improving flexibility during auditory tasks in a controlled way on the one hand, while acknowledging the preferences for visual stimuli in ASD on the other hand.

One might argue that problems in peripheral information processing are responsible for the findings, however, no evidence of peripheral auditory dysfunction has been found in children with ASD (Gravel et al. 2006) and pitch discrimination even seems enhanced (Bonnel et al. 2010). Both computer tasks used in the present study were comparable, thus it is not likely that different task demands are responsible for these results. Taken together, it is then possible that the higher order auditory information is processed in a different way in individuals with ASD (O’Connor 2012), although more research in this area is needed.

Previous studies often found difficulties on visual set shifting tasks in ASD, although the results are not consistent (see for reviews: Geurts et al. 2009, 2014). The differences in results may in part be caused by the multiple functions that are measured by cognitive control tasks. Here, flexibility measurements were controlled for basal attention and response speed but our tasks differed in terms of response type (motor, verbal or paper-and-pencil response) and in terms of task administration (computer vs personal). Although it has been found that individuals with ASD perform better on a computerized version of the WCST test than during administration by a person (Ozonoff 1995), in our study a computer task revealed more difficulties in the ASD group than the paper-and-pencil tasks.

The differences in results may also be caused by the different types of cognitive control tasks used. When only looking at studies in which exactly the same, or highly comparable, computer tasks were used as in the present study, the differences in findings remain puzzling. Two studies found significant differences between individuals with and without ASD (Barneveld et al. 2013; Solomon et al. 2008), while another study did not (Oerlemans et al 2013). These differences in results cannot easily be explained by insufficient power to detect impairments. The studies with negative results (e.g. Oerlemans and the present study), had much larger samples (resp. *n* = 227 and *n* = 117) than the studies in which significant group differences were found (resp. *n* = 69 and *n* = 63, Barneveld et al. 2013; Solomon et al. 2008). The auditory set-shifting tasks were not used in the studies using the same ANT computer test battery (Barneveld et al. 2013; Oerlemans et al. 2013).

A recent meta-analysis of 41 inhibitory control studies confirmed the considerable variability between studies in ASD. The authors argue that, in addition to variability in task conditions, the variability of ASD symptomatology and symptom severity may contribute to the inconsistent findings. They suggest to examine the association between ASD symptom severity and cognitive control performance (Geurts et al. 2014). In this study, symptom severity measured with the SRS was indeed significantly correlated with cognitive control deficits of visual and auditive tasks.

With respect to visual inhibiting a response (stop) and switching (change), no significant group differences were found. However, these visual stop and change tasks correlated with repetitive behavior in the ASD group. In contrast, while the ASD group showed significant slower stopping and changing behavior in response to auditory stimuli than controls, this behavior was in the ASD participants not associated with attention switching in daily life when age was taken into account. Only when including the whole sample (ASD and controls) inhibition in response to auditory stimuli was associated with reported attention switching skills in daily situations. This may be due to insufficient power to detect an association in the ASD group only.

Equal performance between individuals with ASD in comparison with matched controls on the DF task is in line with the study of Kleinhans et al. (2005). Performance on the DF may depend more on fluency and visual scanning than on attentional flexibility (Suchy et al. 2010).

The current study has some limitations. First, it cannot be excluded that statistical corrections for multiple testing may have caused that some differences may not have been picked up (type-2 error), even though the present study is one of the larger that has been published so far. Second, several data were negatively skewed, meaning that lower values (or higher levels of performance) were over-represented. Third, cognitive tasks do not resemble the challenges in daily life. The tasks that were used in the present study enabled us to explore processes that play a role in RRB and to attempt to detangle response inhibition and attentional flexibility. The results suggest, that both processes are highly correlated and both play a role in RRB behavior in daily life. Fourth, we cannot rule out that fatigue might have played a role since all tasks were administered in one session. However, no differences between groups were found with respect to task performance over time, suggesting that fatigue effects, even if present, do not explain the observed differences between ASD patients and control subjects. Fifth, part of the subjects received pharmacological treatment, which may have some effects on our data. However, no differences were observed in tests performance between treated and non-treated subjects. Sixth, we found a deficit in only one task. Interesting though is the finding that deficits exist in the auditory task, a finding that is relatively new and needs replication. It is also important to mention the fact that our study was not designed to predict a three-way interaction between condition, diagnostic group and age group. We are not sure whether these factors interact, or whether they should be viewed as separate phenomena. In order to address this issue, additional studies are required that specifically look at these effects over a much larger span of life with continuous data.

Despite these shortcomings, this study has methodological advantages. The sample was large enough to allow the division in the two age groups and study age related effects. The main reason for this was that our age variable showed as binominal distribution, with a pre puberty and puberty group. During puberty biological changes are huge, also in brain development. The effects of these changes are mostly not linear in nature and are highly complex (Giedd et al. 1999). Another strength is the use of tasks that were comparable in terms of task-conditions but different in terms of information that has to be processed. Most response inhibition or attentional flexibility tasks rely on visual information processing. In the present study an auditory task was added to the test battery which made it possible to compare cognitive responses to visual and auditory information.

## Conclusion

The results of the present study support previous findings of difficulties in both prepotent response inhibition and attentional flexibility in high-functioning children and young adults with ASD in comparison with typically developing control individuals. When controlled for basal attention and response speed, individuals with ASD were slower when asked to inhibit a response or to react in a flexible way to auditory information. It may be important to take that into account when guiding individuals with ASD in everyday situations.
